# Porcelain Inlays

**Published:** 1902-09

**Authors:** Edward C. Briggs

**Affiliations:** Boston, Mass.


### PORCELAIN INLAYS.[1]

BY EDWARD C. BRIGGS, D.M.D., BOSTON, MASS.

Although I am down on the list as reading a paper on "Porcelain Inlays," to be followed by other gentlemen in contributions upon the subject, and would therefore be expected to give something more than a record of practice, nevertheless I shall not go into the history of inlays, but shall make my paper merely a record of personal experience. All of us who have been in practice a score or more of years have made during that time a greater or less number of inlays, and, while the method of grinding out inlays from porcelain teeth or fitting the somewhat crude bits baked from plaster impressions was laborious, and took a great deal of time, I think we all have felt that in the cases where it was tried it paid and was well worth all the trouble. Now, I am not going to pose as a perfectly successful operator in porcelain inlays. I have had failures, plenty of them,—who has not, with whatever material he may use?—but I feel that the aim sought for and the results often reached fully justify one in working along these lines, ever striving to become more and more perfect. In other words, the principle seems, to the writer, to be right.

What Dr. Jenkins has done for us is to make the method easier and of more universal application, without sacrificing the naturalness of the finished product, as has been the case with other confusing enamels.

As before intimated, porcelain inlays or porcelain fillings are not new to the profession, and the writer has personal knowledge of successful ones of fifteen and twenty years' standing. When *properly* and *successfully* made, by whatever method, they have stood the test of time, restoring contours, retaining the natural appearance of the teeth, and preserving them from decay as well as any other filling-material. As compared with such fillings, supposing them to be perfect, Dr. Jenkins's inlays offer no advantages except in the method of producing, which method is made easy by his having invented, or, rather, compounded, an enamel which while retaining the appearance of natural teeth can yet be

---

[1] Read before the American Academy of Dental Science, Boston, Mass., March 5, 1902.

fused by a low heat insufficient to melt thin gold. And even here, with Dr. Jenkins's system, we can get both good and bad results, and practice and experience count for as much as they do in other matters.

In taking impressions one must see to it that the cavity will "draw" well, as you would in making any die. This is common sense, no one man's method. Any contact of the burnisher with the gold tends to draw it away from the tooth (a helpful fact in releasing the gold from the cavity in some cases), and Dr. Jenkins's direction to use punk between burnisher and gold is a most valuable one. Having the impression, we next select the color; and here, of course, one gains by experience, and we learn that in approximal cavities the color selected should be a little lighter than the natural tooth, except where there is a thin enamel wall also exposed to view under which the cement will lie; that in labial cavities the porcelain should be a shade lighter than the tooth in long-lipped people, deeper in color in short-lipped people; that the cement we use changes the whole aspect of things according as it is light or dark.

Then comes the bedding of the gold impression in powdered asbestos, careful that each portion of the gold is supported by the asbestos, and then the fusing. Much has to be learned here. Too rapid fusing makes the inlay porous, and then woe to you if you by any chance grind off the outside glaze. Too long fusing bleaches out the color. In gas furnaces you may gas the filling. All these conditions are common to all porcelain work, and not the special property of the Jenkins enamel. The Jenkins enamel is unquestionably weaker than higher-fusing porcelain, but except in corners of very thin incisors a Jenkins inlay properly baked, the glaze not ground off in any part, and of reasonable thickness, will stand all the wear and tear that may be put upon it.

As to the means of fusing, one can succeed equally well with the Jenkins gas, the Ash electric, or the Hammond electric furnace, and, I presume, many others.

We are now ready to set the inlay, and if thick enough to admit it (and it should be), a groove should be cut on two sides to help in the grip of the cement. The writer has used chiefly Ash's and Howard cement, although the inlays of a dozen years ago were set in Weston's insoluble cement.

Of course, the porcelain is to be tried in, and if necessary ground to suit the bite or to make the edges flush with the tooth. This grinding is, however, to be deplored, as it too often discloses a fatal porosity, while it is possible to produce inlays with a very small per cent. of them requiring grinding and fitting. Having smeared the surface of the cavity and the inside of the porcelain with thin but *thoroughly mixed* cement, we push the inlay to place with a soft wood stick, using all the pressure one is capable of. And now let us be patient and wait for the cement to harden, realizing that a half-hour now is worth years in the security of the joint. If the rubber dam is on, one can tie a string around it close to the cutting edges of the teeth, and then, cutting the dam off at the string, have the tooth in a neat rubber bag, with which the patient can comfortably wait in the reception-room or even go about his business, pulling off the rubber at a stated time.

To what are the failures due? Some cases have been referred to, but there will be no harm if we repeat. In the first place, we have the shape of the cavity to consider, and it is not fair to expect a porcelain inlay, even with the adhesiveness of the cement, to stick in a cavity where you would not expect gutta-percha to stay. While your matrix impression must draw well from the cavity, yet the cavity should be something more than a concave depression; and, with a clear eye to the "drawing," one must at the same time make a good square-edged, dependable cavity. Another cause or causes of failure are the disasters in fusing, before alluded to, and common to all porcelain work. Then, of course, the cement must be good, must be properly mixed (and it is surprising how few know how to mix cement), and be allowed to thoroughly harden.

The edge-strength is good if you allow it to have something better than a knife edge.

Bubbles will, of course, sooner or later disclose themselves.

There is no position of a cavity that may not be filled with porcelain, and I recall one patient who has twenty-one porcelain fillings. This patient presented herself with teeth badly broken down, filled with cement, and the pulps for the most part alive.

I have used other bodies, higher fusing, which, of course, give greater strength, as Whitely, Parker, Ash, but have never been able, except with Parker's, which ruins all my furnaces, to get a very natural appearance.

The records in my office show that we have made over two thousand Jenkins porcelains, and the failures have been only about one per cent., due to unwise shaping of cavities, hurrying the setting of the cement, or grinding off the glaze in adjusting the bite, —all avoidable errors.

Properly made and properly set, porcelain fillings present a most satisfactory appearance, preserving the teeth as well as, if not better than, any other material we use.

